# Gas exchange and hydraulics during drought in crops: who drives whom?

**DOI:** 10.1093/jxb/ery235

**Published:** 2018-07-18

**Authors:** Jaume Flexas, Marc Carriquí, Miquel Nadal

**Affiliations:** Research Group in Plant Biology under Mediterranean Conditions, Universitat de les Illes Balears-Instituto de Agroecología y Economía del Agua (INAGEA), Palma, Illes Balears, Spain

**Keywords:** Drought stress, gas exchange, leaf hydraulics, mesophyll conductance, photosynthesis, stomatal conductance

## Abstract

This article comments on:

Wang X, Du T, Huang J, Peng S, Xiong D. 2018. Leaf hydraulic vulnerability triggers the decline in stomatal and mesophyll conductance during drought in rice (*Oryza sativa*). Journal of Experimental Botany **69,** 4033–4045.

**The correlation between stomatal, mesophyll and leaf hydraulic conductance (*K*_leaf_), and the timing of each during regulation under drought, are not fully understood. Studies which make precise, parallel measurement of these variables during progressive imposition of drought are needed.** **Wang *et al.* (2018)** **provide novel insights, showing that, in rice, a decline of *K*_leaf_ is the earliest response to decreasing water availability, and they propose that it triggers the later decline of stomatal and mesophyll conductance. Comparison with results from other species intensifies the debate about the relationships between these variables, as well as between photosynthesis (i.e. productivity) and hydraulic failure (death).**

Drought stress is one of the largest threats to crop productivity and survival worldwide (Boyer, 1982; Ciais *et al.*, 2005), hence the importance of unveiling the relationships between the different physiological mechanisms and traits that confer resistance in plants (McDowell *et al.*, 2013). Water stress causes the decrease in leaf water potential (Ψ_leaf_), which in turn causes the activation of turgor-related signals (Rodriguez-Dominguez *et al.*, 2016) and/or hormonal signals. Abscisic acid (ABA) is considered the main plant hormone involved in the water stress response, although there is still debate as to whether the fraction of the total hormone pool involved in signalling is synthesized mostly in the roots (Dodd, 2005) or in the same leaf (McAdam *et al.*, 2016). These hydraulic and non-hydraulic factors regulate stomatal but apparently also mesophyll conductances to control both transpiration (i.e. reduce hydraulic tension in the atmosphere–plant–soil continuum) and CO_2_ supply for the optimization of gas exchange (Nadal and Flexas, 2018). These signals are coupled with the supply capacity of the hydraulic system, otherwise extreme water loss and/or hydraulic failure could lead to complete desiccation of the plant (Sperry, 2004; Hochberg *et al.*, 2017). However, this general scheme of drought response may vary between plants depending on the degree of iso- or anisohydry (Martínez-Vilalta and Garcia-Forner, 2017). Signals induced by Ψ_leaf_ also regulate leaf hydraulic conductance (*K*_leaf_) (Coupel-Ledru *et al.*, 2017), in tight coordination with gas exchange (Brodribb *et al.*, 2014; Gleason *et al.*, 2017). Decreases of *K*_leaf_ are generally associated with hydraulic failures, such as embolism, but also with other forms of regulation (Hochberg *et al.*, 2017). However, the relative importance and mechanisms of regulation of its components – the conductance within the xylem (*K*_x_) and the outside-xylem conductance (*K*_ox_) – during drought remain unresolved (Trifiló *et al.*, 2016). If the drought worsens, the physiological effects on the leaves are incrementally increased, which may lead to the death of the leaf (e.g. full hydraulic failure, or 100% embolism; Martin-StPaul *et al.*, 2017), and the whole plant may depend on the existence of safety margins among plant organs (Liu *et al.*, 2015; Skelton *et al.*, 2017; Rodriguez-Dominguez *et al.*, 2018). Although the main processes that occur during drought are clear, knowledge of the general timescale of response and the importance of each parameter is limited because most studies do not monitor the same variables simultaneously, and few consider so many parameters during a prolonged drought as do Wang *et al.* (2018). So what do we really know about these inter-relationships and why is the work by Wang *et al.* important?

## Variability in the physiological responses of crops to drought stress

There are very few interspecific studies on limitations to photosynthesis under drought, thus precluding broad generalizations. For instance, although a pattern has been suggested in which diffusion conductances limit photosynthesis under mild and moderate stress, while biochemical limitations appear only at the later stages (reviewed in Nadal and Flexas, 2018), some studies have found differences among species, especially regarding the relative importance of stomatal and mesophyll limitations (Galmés *et al.*, 2007; Flexas *et al.*, 2009; Galle *et al.*, 2011) but also concerning the early appearance of biochemical limitations (Ennahli and Earl, 2005). Similarly, while it seems that a general coordination among both conductances occurs during drought, recent studies suggest that the nature of the relationship may be species-specific. In this sense, Flexas *et al.* (2013*a*) showed that the relationship between *g*_s_ and *g*_m_ varies across crops under well-watered and water-stressed conditions: although most of them show a tight coordination between these two conductances, some (e.g. poplar) did not show such relationship.

In two rice cultivars, Wang *et al*. show that there is strong coordination between *K*_leaf_, *g*_s_ and *g*_m_ during their decrease under drought. Indeed, a similar sequence of events can also be observed for olive when combining data from several studies (Box 1), although olive seems to operate along a wider range of Ψ_leaf_. On the other hand, this early decline in all three conductances is not observed in grapevine, where the decline of *K*_leaf_ (*P*_50_) occurs at the latest stages of water stress, after a previous progressive and strong decrease in photosynthesis, mainly due to limitation by stomatal conductance. The three examples displayed in Box 1 suggest different possibilities regarding limitations to photosynthesis and coordination of conductances across species.

**Box 1. Limitations to net assimilation in relation to the vulnerability of its constraints (*g*_s_, *g*_m_, biochemistry and *K*_leaf_) in different crops**

Response of limitations to photosynthesis – stomatal (SL), mesophyll conductance (ML) and biochemical (BL) limitations – to decreasing leaf water potentials (Ψ_leaf_) in *Oryza sativa* (Wang *et al.*, 2018), *Olea europaea* (data combined from Perez-Martin *et al.*, 2009, and Varone *et al.*, 2012) and *Vitis vinifera* (from El Aou-ouad *et al.*, 2016). *K*_leaf_*P*_50_ and *P*_80_ are represented by red dashed and solid lines (data from Wang *et al.*, 2018, for rice, and data combined from Torres-Ruiz *et al.*, 2015, and Hernandez-Santana *et al.*, 2016, for *O. europaea*, and from Martorell *et al.*, 2015, for *V. vinifera*). Yellow points in *O. sativa* represent the *P*_50_ of *g*_s_, *g*_m_ and electron transport rate (ETR) (each of them situated over the upper line of its limitation – SL, ML or BL, respectively – data from Wang *et al.*, 2018). The blue dotted line represents the turgor loss point (data from Wang *et al.*, 2018, for rice, and value from Hernandez-Santana *et al.*, 2016, for *O. europaea* and from Martorell *et al.*, 2015, for *V. vinifera*). The orange dotted line accounts for either *K*_x_*P*_50_ in *O. sativa* (value from Stiller *et al.*, 2003) or the Ψ_leaf_ in which approximately 50% embolism occurs in the leaf midrib (based on optical measurements; data from Rodriguez-Dominguez *et al.*, 2018, for *O. europaea* and from Hochberg *et al*., 2017 for *V. vinifera*).

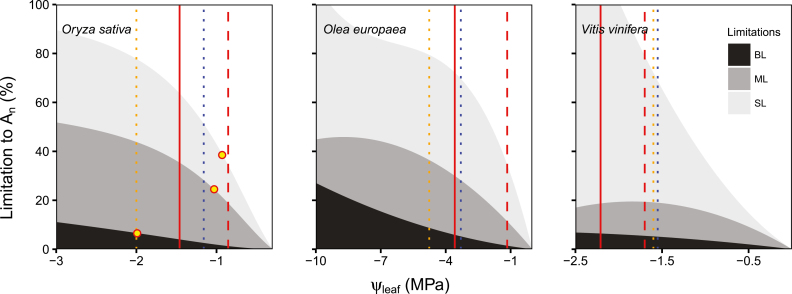

The species-dependent coordination between stomatal and *K*_leaf_ responses to drought could indicate different strategies regarding water conservation and safety of transport (see Box 2). As shown by Wang *et al*., rice presents a tight coordination between *K*_leaf_ and *g*_s_; in fact, the decrease of *g*_s_ is mainly attributed to *K*_leaf_. This has also been shown in woody crops (Hernandez-Santana *et al.*, 2016; Rodriguez-Dominguez *et al.*, 2016). On the other hand, no such coordination has been observed in soybean (Locke and Ort, 2014). On a broader phylogenetic scale, clearer differences emerge; for example, *g*_s_ presents a higher sensitivity to Ψ_leaf_ in ferns compared to coexisting angiosperms (Brodribb and Holbrook, 2004). In ferns, stomata closed before any significant drop in *K*_leaf_, whereas in the angiosperms studied there was a tighter coordination between *g*_s_ and *K*_leaf_. This was also observed when studying the different responses of *g*_s_ and *K*_leaf_ not to drought but to varying light intensity (Xiong *et al.*, 2018). Indeed, the differences in *P*_50_ for *g*_s_ and *K*_leaf_ may be more related to phylogeny than to ambient conditions as no common pattern in *P*_50_ was observed in co-occurring tree species (Liu *et al.*, 2015). In the case of the drought-induced *g*_m_–*K*_leaf_ relationship, significant variability has been reported even at the clone level (Théroux-Rancourt *et al.*, 2015). Some degree of plasticity in these relationships has also been seen in grapevines, where *K*_leaf_ presented a decreasing *P*_80_ as summer progressed (Martorell *et al.*, 2015). Moreover, even the mechanistic basis for the decline in *K*_leaf_ (i.e. the relative importance of *K*_ox_ and *K*_x_) may be species-dependent (Trifiló *et al.*, 2016). All these examples of interspecific variation hinder disentanglement of the factors limiting photosynthesis and transpiration under water stress.

**Box 2. Interrelationships between stomatal and hydraulic conductance in different crops**

The graph shows the relationships between stomatal (*g*_s_) and leaf hydraulic (*K*_leaf_) conductances and the magnitudes of each for the same crop species considered in Box 1: *Oryza sativa* (mean data from Wang *et al.*, 2018), *Olea europaea* (data combined from Fernandes-Silva *et al.*, 2016; Hernandez-Santana *et al.*, 2016) and *Vitis vinifera* (data combined from Pou *et al.*, 2012, 2013; El Aou-ouad *et al.*, 2017). Lines represent quadratic polynomial fittings for each species and shaded areas are their 95% confidence intervals.

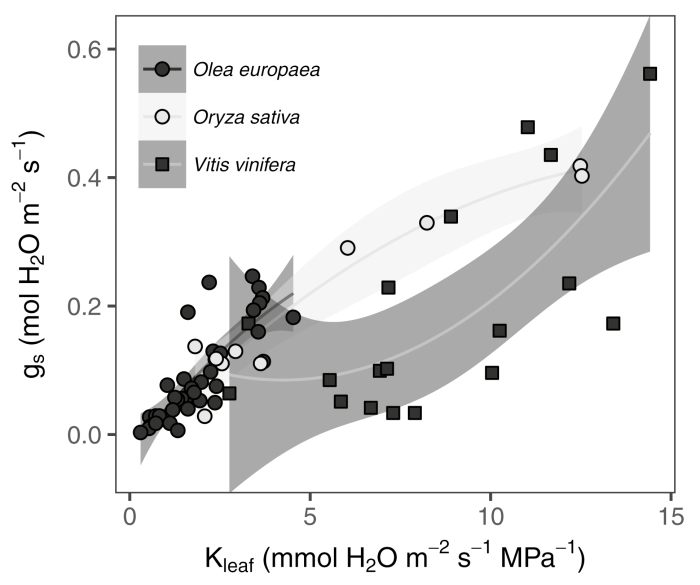

## Role of, and relationships among, water conductances during drought: universal or species-specific?

Many theories have considered the stomata as the safety valves preventing hydraulic dysfunction under mild to moderate water stress conditions (Hochberg *et al.*, 2017 and references therein), considering leaf xylem hydraulic vulnerability as the main component of leaf hydraulic vulnerability. However, results from Wang *et al.* challenge these theories. The fact that the *K*_leaf_*P*_50_ was achieved before the *g*_s_ and *g*_m_*P*_50s_ suggests that, in rice, the stomata do not function as a safety valve and therefore either: (i) if *K*_leaf_=*K*_x_, leaf xylem cavitated before stomata closed; or (ii) if *K*_leaf_=*K*_ox_, outside-xylem hydraulic vulnerability protected against xylem failure instead of stomata (see Box 3 for a depiction of these two possibilities). The first hypothesis is unlikely as the xylem vulnerability *P*_50_ reported by Stiller *et al.* (2003) is about –2.0 MPa. On the other hand, although Wang *et al.* measured *K*_leaf_ without distinguishing *K*_x_ from *K*_ox_, the second hypothesis may be more likely: indeed, Trifiló *et al.* (2016) and Scoffoni *et al.* (2017) showed that outside-xylem hydraulic vulnerability explains 75 to 100% of *K*_leaf_ decline before reaching the turgor loss point in most of the species studied. However, this hypothesis cannot be considered confirmed yet, at least for all vascular plants, as measurements performed using new techniques (such as the leaf optical vulnerability; Brodribb *et al.*, 2016) that allow the simultaneous measurement of *K*_x_ and *g*_s_ (Hochberg *et al.*, 2017) provide new evidence supporting the hypothesis that *K*_leaf_ is mainly driven by *K*_x_. Nonetheless, these two hypotheses are not necessarily irreconcilable; in fact, they may represent species- or even genotype-specific strategies for plants coping with water stress along the iso–anisohydric spectrum (Tombesi *et al.* 2014; Coupel-Ledru *et al.*, 2017).

**Box 3. Variables and hypothetical relationships controlling physiological drought response in crops**

Diagram showing the potential interrelations between water potential and leaf conductances under mild to moderate drought stress conditions. Solid lines indicate positive relationships between variables, whereas broken lines indicate negative relationships. The dotted broken line indicates the hydraulic disconnection between leaf and stem due to embolism. Left diagram (a) follows the hypothesis of the safety valve function of stomata to prevent hydraulic failure (*K*_leaf_ mostly constituted by leaf xylem conductance, *K*_x_). In this scenario, stomatal and mesophyll conductance (*g*_s_ and *g*_m_, respectively) are reduced to keep *K*_leaf_ within the safety margin to avoid hydraulic disconnection from the stem. Right diagram (b) reflects a hypothesis that can be derived from the suggestion by Wang *et al.* of outside-xylem conductance controlling *K*_leaf_, which in turn triggers the decline of both *g*_s_ and *g*_m_. In this case, cavitation would be of little magnitude because *K*_leaf_ would be governed mainly by *K*_ox_. Notice the double-arrowed blue line linking *g*_m_ and *K*_leaf_ in both diagrams; this accounts for the coordinated nature of these two conductances (Flexas *et al.*, 2013*b*), which could emerge from a common structural basis (Xiong *et al.*, 2017), rather than by one being directly affected by the other.

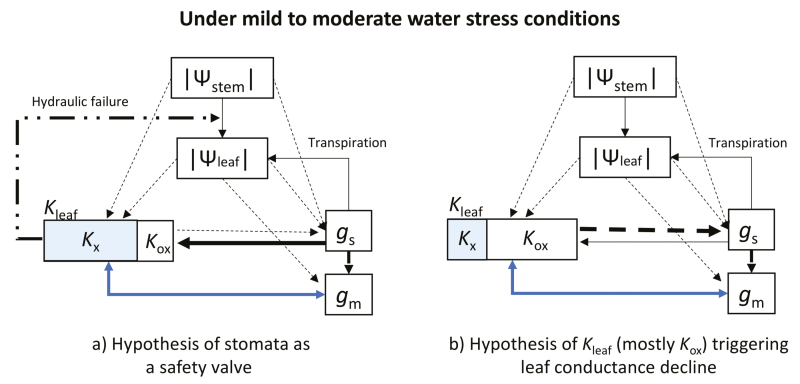

In summary, until methodological limitations are improved, and more experiments are carried out monitoring the multiple interrelated variables that act during drought for multiple species, a very interesting debate where (at least) two major hypotheses are possible will continue. The work by Wang *et al.* (2018) adds important new data and ideas to this debate.
